# ML364 exerts the broad-spectrum antivirulence effect by interfering with the bacterial quorum sensing system

**DOI:** 10.3389/fmicb.2022.980217

**Published:** 2022-12-22

**Authors:** Youwen Zhang, Limin Dong, Lang Sun, Xinxin Hu, Xiukun Wang, Tongying Nie, Xue Li, Penghe Wang, Pengbo Pang, Jing Pang, Xi Lu, Kaihu Yao, Xuefu You

**Affiliations:** ^1^Beijing Key Laboratory of Antimicrobial Agents, Institute of Medicinal Biotechnology, Chinese Academy of Medical Sciences and Peking Union Medical College, Beijing, China; ^2^Key Laboratory of Major Diseases in Children, Ministry of Education, National Key Discipline of Pediatrics (Capital Medical University), Beijing Pediatric Research Institute, Beijing Children’s Hospital, National Center for Children’s Health, Capital Medical University, Beijing, China

**Keywords:** ML364, autoinducer-2, antimicrobial resistance, broad-spectrum antivirulence agent, quorum sensing

## Abstract

Antivirulence strategy has been developed as a nontraditional therapy which would engender a lower evolutionary pressure toward the development of antimicrobial resistance. However, the majority of the antivirulence agents currently in development could not meet clinical needs due to their narrow antibacterial spectrum and limited indications. Therefore, our main purpose is to develop broad-spectrum antivirulence agents that could target on both Gram-positive and Gram-negative pathogens. We discovered ML364, a novel scaffold compound, could inhibit the productions of both pyocyanin of *Pseudomonas aeruginosa* and staphyloxanthin of *Staphylococcus aureus*. Further transcriptome sequencing and enrichment analysis showed that the quorum sensing (QS) system of pathogens was mainly disrupted by ML364 treatment. To date, autoinducer-2 (AI-2) of the QS system is the only non-species-specific signaling molecule that responsible for the cross-talk between Gram-negative and Gram-positive species. And further investigation showed that ML364 treatment could significantly inhibit the sensing of AI-2 or its nonborated form DPD signaling in *Vibrio campbellii* MM32 and attenuate the biofilm formation across multi-species pathogens including *Pseudomonas aeruginosa*, *Escherichia coli*, *Klebsiella pneumoniae* and *Staphylococcus aureus*. The results of molecular docking and MM/GBSA free energy prediction showed that ML364 might have higher affinity with the receptors of DPD/AI-2, when compared with DPD molecule. Finally, the *in vivo* study showed that ML364 could significantly improve the survival rates of systemically infected mice and attenuate bacterial loads in the organs of mice. Overall, ML364 might interfere with AI-2 quorum sensing system to exert broad-spectrum antivirulence effect both *in vitro* and *in vivo*.

## Introduction

An emerging public health threat of the 21st century is bacterial antimicrobial resistance (AMR), caused by changes in bacteria that make antibiotics no longer effective. In 2019, there were approximately 4.95 million deaths related to bacterial antimicrobial resistance, based on the systematic analysis conducted by Antimicrobial Resistance Collaborators (Murray et al., 2022). *Escherichia coli*, *Staphylococcus aureus*, *K. pneumoniae*, *Streptococcus pneumoniae*, *Acinetobacter baumannii*, and *Pseudomonas aeruginosa* were the six pathogens responsible for the majority of deaths caused by resistance in 2019. WHO has classified pathogens into three categories (critical, high, and medium priority) to guide discovery, research, and development of new antibiotics for drug-resistant bacterial infections. Critical-priority bacteria included carbapenem-resistant *Acinetobacter baumannii, Pseudomonas aeruginosa*, and carbapenem-resistant and third-generation cephalosporin-resistant Enterobacteriaceae. The highest ranked Gram-positive bacteria (high priority) were vancomycin-resistant *Enterococcus faecium* and methicillin-resistant *Staphylococcus aureus* (Tacconelli et al., 2018; Murray et al., 2022). But unfortunately, few new scaffold antibiotics against these priority pathogens have been involved in the pipeline for the last decades due to sluggish antibiotic discovery and development (Theuretzbacher et al., 2020).

Antivirulence strategy has been developed as a nontraditional therapy which would engender a lower evolutionary pressure toward the development of antibiotic resistance. However, the majority of the antivirulence agents currently in development could not meet clinical needs due to their narrow antibacterial spectrum and limited indications (Theuretzbacher and Piddock, 2019). For now, two classes of agents have been developed for broadening the antibacterial spectrum of antivirulence strategy. One is liposome which could neutralize the toxins generated by pathogens. The first-in-human clinical trial of the liposome CAL02 developed by Combioxin company was accomplished in the year of 2020 (Laterre et al., 2019). The other class is the small molecule which could interfere with the quorum sensing system of pathogens. For instance, LED209, a potent small molecule inhibitor of the QseC receptor, could disrupt the sensing of bacterial AI-3 signaling and prevent the activation of the virulence program of several Gram-negative pathogens both *in vitro* and *in vivo* (Curtis et al., 2014). Moreover, the coumarin family compounds could block Gram-negative bacteria’s classical AHL-based system *in vitro*, by particularly against short, medium, and long chain N-acyl-homoserine lactones (Gutierrez-Barranquero et al., 2015). Although some of these compounds could inhibit the virulence across multi-species of bacteria, the pathogens they targeted were still limited to either Gram-negative or Gram-positive bacteria. Few of them could exert the antivirulence effect on both Gram-negative and Gram-positive pathogens.

Due to the virulence factor pigments generated by *P. aeruginosa* and *S. aureus* were easy to observe, we chose *P. aeruginosa* and *S. aureus* as the representative species for Gram-negative and Gram-positive bacteria to screen the potential broad-spectrum antivirulence agents from our in-house compound library. Fortunately, we discovered ML364, a novel scaffold small molecule, could alleviate the pyocyanin and staphyloxanthin production *in vitro*. ML364 is an inhibitor of ubiquitin specific peptidase 2 (USP2) in human breast cancer (Davis et al., 2016). ML364 could increase cellular cyclin D1 degradation and cause cell cycle arrest in cancer cell lines. Furthermore, pyocyanin and staphyloxanthin were the main virulence factor pigments produced by *P. aeruginosa* and *S. aureus*, respectively (Ni et al., 2020). Moreover, the further transcriptome sequencing data showed that the quorum sensing system of pathogens was mainly disrupted by ML364 treatment. And to date, autoinducer-2 (AI-2) of the QS system is the only non-species-specific signaling molecule identified that is produced by many bacteria including both Gram-negative and Gram-positive species (Pereira et al., 2013). Finally, we discovered that ML364 could interfere with AI-2 quorum sensing system and inhibit biofilm formation across multi-species pathogens. (*S*)-4,5-dihydroxypentane-2,3-dione (DPD), the nonborated form of AI-2, could spontaneously interconvert into AI-2 signaling molecules (Zhang et al., 2020). Further molecular docking and MM/GBSA (molecular mechanics/generalized Born surface area) free energy calculation showed that ML364 had higher affinity with the receptors of DPD/AI-2, when compared with DPD. And the *in vivo* study showed that ML364 could significantly improve the survival rate of systemically infected mice and attenuate the bacterial loads in the organs of systemically infected mice. In conclusion, ML364 could exert the broad-spectrum antivirulence effect both *in vitro* and *in vivo* by interfering with the bacterial quorum sensing system.

## Materials and methods

### Bacterial strains and culture conditions

In this study, carbapenem-resistant *P. aeruginosa* (CRPA) 16-2, methicillin-resistant *S. aureus* (MRSA) 08-50 and any other clinical isolates were obtained from the Chinese Academy of Medical Sciences (CAMS-CCPM-A). In addition, *P. aeruginosa* PAO1, *S. aureus* ATCC29213 and any other ATCC reference strains were obtained from the American Type Culture Collection (ATCC). We routinely cultured the isolates in Tryptic Soy Broth (TSB), Luria-Bertani (LB) broth, or cation-adjusted MH broth (CAMH) under freezing conditions at −80°C.

### Antimicrobial susceptibility testing

All antibiotics were obtained from the National Institutes for Food and Drug Control (Beijing, China). ML364 was purchased from Target Molecule (Boston, MA, United States). Based on guidelines established by the Clinical and Laboratory Standards Institute (M100-S31), the antibiograms for ML364 and levofloxacin were determined by agar dilution. 16–20 h at 37°C were required for incubation of the agar plates. Checkboard assay was conducted as previously described (Zhang et al., 2019). Briefly, 1–64 μg/ml meropenem were mixed with 8–512 μg/ml ML364, or 16–1,024 μg/ml methicillin were mixed with 1–64 μg/ml ML364. In 96-well plates, two combinations of drugs were mixed, then a bacterial suspension was added at a final concentration of 5 × 10^5^ CFU/ml in CAMH broth. Following 18 h incubation period at 37°C, results were observed. Three replicates of each experiment were performed on separate days.

### Growth curve analysis

Four independent cultures of *P. aeruginosa* and *S. aureus* were grown overnight and then diluted 1,000 times with CAMH broth. *P. aeruginosa* PAO1 and CRPA 16-2 were treated with 64–256 μg/ml ML364, and *S. aureus* ATCC 29213 and MRSA 08-50 were treated with 8–64 μg/ml ML364. A culture of 0.3 ml was added to the Bioscreen C plate (Sun et al., 2021). A Bioscreen C reader (FP-1100-C; Oy Growth Curves Ab Ltd.) was used to measure optical density at 600 nm every 10 min when strains were cultured in CAMH broth with or without ML364 at 37°C for 24 h. Growth curves were determined by evaluating the OD_600nm_ values throughout the 24 h.

### Scanning electron microscopy

The overnight cultures (1 ml) of *P. aeruginosa* PAO1 with and without 256 mg/ml ML364 or *S. aureus* ATCC 29213 with and without 64 μg/ml ML364 were centrifuged for 3 min at 6,000 ×g. The bacterial pellets were washed twice in phosphate-buffered saline before resuspended in 1 ml of phosphate-buffered saline after discarding the supernatants. Fixation was carried out at 4°C with glutaraldehyde 2.5% overnight. Then the fixative was removed by centrifugation at 6,000 ×g for 3 min. Image analysis was performed using a scanning electron microscope (Hitachi SU8020, Tokyo, Japan).

### Virulence factor quantification assay

In order to determine the concentration of pyocyanin, *P. aeruginosa* PAO1 and carbapenem-resistant *P. aeruginosa* (CRPA) 16-2 were cultivated in glycerol-alanine medium with or without ML364 at 37°C with shaking for 24 h (Frank and Demoss, 1959). Cells were mixed with chloroform and centrifuged after mixing. After extracting 1 ml of the chloroform layer, it was mixed with the same volume of 0.2 mol/l HCl (Essar et al., 1990). Perkin Elmer EnSpire Multilabel Plate Reader (HORIBA ABX) was used to measure the OD_520nm_ of the pink pigment. Staphyloxanthin was quantitated using the methanol extraction method described previously (Ni et al., 2017; Pandey et al., 2019). Briefly, *S. aureus* ATCC 29213 and methicillin-resistant *S. aureus* (MRSA) 08-50 were cultured in TSB broth for 48 h, either with or without ML364. A total of 3 ml of bacterial cultures was centrifuged and washed. After incubating for 10 min in a 58°C dry block bath coupled with intermittent vortexing, the pellet was diluted with 0.5 ml of methanol. To adjust bacterial cell absorbance by OD_600nm_, the OD_450nm_/OD_600nm_ of the supernatant was determined.

### Transcriptomic analysis

At 37°C with constant shaking, *P. aeruginosa* PAO1 was cultured in LB broth with and without 256 μg/ml ML364 for 6 h. A TianGen RNAprep Pure Kit (TianGen) was used to harvest the cells for RNA extraction. We nurtured *S. aureus* ATCC 29213 in TSB broth treated with and without 64 μg/ml of ML364 for 6 h at 37°C. A total of 10 μl of lysostaphin (10 mg/ml) was applied for bacteriolysis. Cells were harvested for RNA extraction by employing the RNA extraction kit. Three biological replicates were used in the experiment. Subsequently, after the integrity was assessed, a total of 3 μg RNA was used to generate sequencing libraries. After cluster generation, the library preparations were sequenced. DESeq2 R package (1.20.0) was used to analyze differential expression between two groups. We defined a threshold for significantly differential expression as *p* < 0.05 and absolute log_2_ (foldchange) > 0. And KEGG (Kyoto Encyclopedia of Genes and Genomes) enrichment analysis of differentially expressed genes was conducted *via* KOBAS-i software (Bu et al., 2021).

### Relative gene expression analysis *via* real-time PCR

RNA was extracted from *P. aeruginosa* PAO1 treated with and without ML364 in accordance with the protocols described for transcriptomic analysis (Dong et al., 2019). A FastQuant RT Kit (TianGen) was used to reverse transcribe the mRNA and generate cDNA. The 7,500 Fast Real-Time PCR System (Applied Biosystems, Foster City, CA, United States) was used for all real-time PCR analyses. The PCR reaction mixtures included the following components: Power SYBR Green PCR Master Mix (Applied Biosystems), forward and reverse primer mix (the primer sequences are listed in Supplementary Table 1), template DNA and nuclease-free water. The cycling conditions were 50°C for 2 s, 95°C for 10 min, followed by 40 cycles at 95°C for 15 s and 60°C for 1 min.

### Autoinducer-2 inhibition assay

An overnight culture of *V. Campbellii* MM32 grown in AB medium was diluted 1:2,500 into fresh Autoinducer Bioassay Medium (TOPBIO, Shandong, China), and 100 μl aliquots of the diluted cells were added to 96-well microtiter plates. Subsequently, 100 μl aliquots of ML364 (0.25–64 μg/ml) mixed with the corresponding concentrations of DPD (Omm Scientific, Texas, USA) or autoinducer-2were added to the wells and the microtiter plates were incubated at 30°C for 6 h with shaking at 180 r.p.m. (Jiang et al., 2018). Bioluminescence was measured using Perkin Elmer 2,300 EnSpire microplate reader. Three replicates of each experiment were performed on separate days.

### Biofilm formation assay

The formation of biofilms on 96-pin microtiter plate lids was conducted as described before (Tsukatani et al., 2020). Briefly, an overnight culture of bacteria grown in BHI medium was diluted 1:100 into fresh BHI broth medium. The cell of 96-well microtiter plate was filled with 100 μl bacterial inoculum and 100 μl aliquots of ML364 (64–256 μg/ml for Gram-negative bacteria, 16–64 μg/ml for Gram-positive bacteria). Then, the plate was covered with a 96-pin microtiter plate lid (Nunc-Immuno TSP; Thermo Fisher Scientific Inc., MA, United States) and incubated for 24 h. After incubation, the 96-pin lids with established biofilms were washed three times and then placed onto fresh 96-well microtiter plates with each well containing 200 μl of 0.1% crystal violet solution. The 96-pin lids with biofilms were stained for 0.5 h and then washed three times and positioned on fresh 96-well microtiter plates in which 200 microliter of absolute ethanol was added to each well. After 15 min, the amount of violet extract was quantified at 590 nm by using a microplate reader. To adjust the bacterial cell absorbance, the OD_590nm_ of the violet extract was divided by OD_600nm_ of the bacterial inoculum. The experiments were performed in triplicate on different days.

### *In silico* study

In fastDRH webserver, the AutoDock Vina 1.2.3 docking engine was chosen to generate 15 docking poses of ML364 and DPD (Eberhardt et al., 2021; Wang et al., 2022). Structure-truncated MM/GBSA free energy was calculated to rescore the poses. Ff19SB (with OPC water model) was chosen for the receptor force field, while GAFF2 was chosen for the ligand force field. Protein residues within 8 Å of all ligand binding poses were retained for rescoring. For the per-residue free energy decompositions analyses, the receptor force field and the ligand force field were in accordance with rescoring procedure. Protein residues within 20 Å of all ligand binding poses were retained for free energy calculations by MM/GBSA. Moreover, the residues with interaction energy ≥ 0.1 kcal/mol or ≤ −0.1 kcal/mol were retained for further analyses. ML364 (Pubchem CID: 70789348) and DPD (Pubchem CID: 11170991) were used to dock into the protein TlpQ (PDB: 6FU4), PctA (PDB: 5T7M), and LuxP (PDB: 1JX6). The interactions between proteins and ligands were analyzed by PLIP (Protein-Ligand Interaction Profiler; Adasme et al., 2021) and images were generated using the PyMol Molecular Graphics system v.2.5.2.

### *In vivo* treatment evaluation

The stock solution of ML364 was prepared in 10% ethanol, 10% cremophor, and 80% D5W (5% dextrose in water). All mice (ICR, 18–20 g) were obtained from Beijing Vital River Laboratory Animal Technology Co. Ltd. A dose of 100 mg/kg ML364 was injected intraperitoneally into mice (*n* = 10 per group for both sexes). The changes in body weight were recorded for 1 week after injection. Then female mice (*n* = 10 per group) were infected intraperitoneally with 0.5 ml bacterial suspensions of *P. aeruginosa* PAO1, CRPA 16-2, *S. aureus* ATCC 29213, and MRSA 08-50 in 5% mucin. A dose of 3 mg/kg ML364 was intraperitoneally injected at 0, 12, and 24 h post-infection. Survival rates of mice were monitored until 4 days post-infection. The survival distributions of different samples were compared using the log-rank test.

The female mice were intraperitoneally infected with CRPA 16-2 and MRSA 08-50 to determine the colony counts of different organs in different treatment groups. 3 mg/kg ML364 was intraperitoneally injected at 0, 12, and 24 h post-infection. At 26 h post-infection, aseptically removed spleens, livers, and kidneys were homogenized, diluted, and plated on LB agar to determine CFU counts (*n* = 7 per group). The statistical significance of bacterial loads between various treatment groups was calculated by using one-way ANOVA and Bonferroni’s multiple comparisons.

All animal husbandry and experimentation procedures have been conducted in accordance with Chinese national standards for laboratory animals (GB/T 35892-2018) with approval from the Laboratory Animal Welfare and Ethics Committee of the Institute of Medicinal Biotechnology, Peking Union Medical College.

## Results

### The *in vitro* studies of ML364

**Figure 1:**
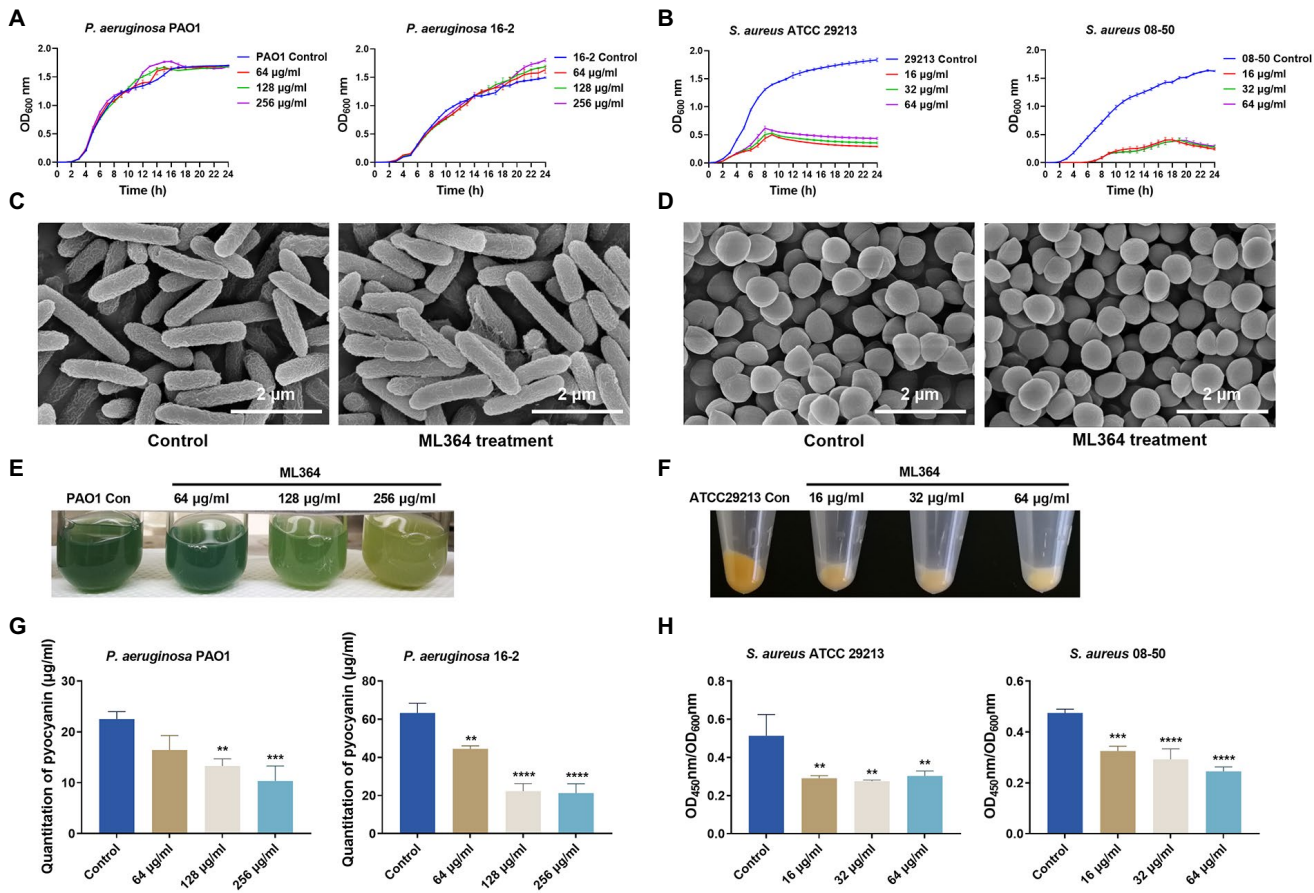
The *in vitro* studies of ML364. **(A)** Growth curves of *Pseudomonas aeruginosa* PAO1 and carbapenem-resistant *P. aeruginosa* (CRPA) 16-2 treated with and without ML364. **(B)** Growth curves of *S. aureus* ATCC 29213 and methicillin-resistant *Staphylococcus aureus* (MRSA) 08-50 treated with and without ML364. **(C)** Images from SEM for *P. aeruginosa* PAO1 treated with or without 256 μg/ml ML364. **(D)** Images from SEM for *S. aureus* ATCC 29213 treated with or without 64 μg/ml ML364. **(E)** Production of pyocyanin by *P. aeruginosa* PAO1 in the presence or absence of ML364. **(F)** Production of staphyloxanthin by *S. aureus* ATCC 29213 in the presence or absence of ML364. **(G)** Quantitation of pyocyanin in *P. aeruginosa* PAO1 and CRPA 16-2 treated with and without 64–256 μg/ml ML364. **(H)** Quantitation of staphyloxanthin in *S. aureus* ATCC 29213 and MRSA 08-50 treated with and without 16–64 μg/ml ML364. Control represents the solvent treatment group. Data were calculated with one-way ANOVA and Bonferroni’s multiple comparisons, compared to those of the control group; ^*^p < 0.05, ^**^p < 0.01, ^***^p < 0.001, ^****^p < 0.0001.

The MICs of ML364 against multiple bacterial species were first determined (Supplementary Table 2). MICs of ML364 for both Gram-negative and Gram-positive bacteria were all ≥512 μg/ml, indicating no bactericidal activity. Respectively, for CRPA 16-2 or MRSA 08-50, 512 μg/ml or 64 μg/ml ML364 showed no synergistic effect when combined with meropenem or methicillin (Supplementary Table 3). Furthermore, the growth curves of *P. aeruginosa* PAO1 and CRPA 16-2 could not be changed by 64, 128, or 256 μg/ml ML364 treatment (Figure 1A). For *S. aureus* ATCC 29213 and MRSA 08-50, although ML364 exerted no bactericidal effect on both strains, the growth curves showed that 16–64 μg/ml ML364 could inhibit the growth of the *S. aureus* strains and result in about 4-fold decrease of OD_600nm_ (Figure 1B). However, on scanning electron microscopy images of *P. aeruginosa* PAO1 or *S. aureus* ATCC 29213, there was no apparent change in the morphology of the ML364-treated cells (Figures 1C,D).

As determined by pigment quantification, ML364 could significantly inhibit pyocyanin and staphyloxanthin synthesis compared to the control group. (*p* < 0.05; Figures 1E,F). The production of pyocyanin in *P. aeruginosa* PAO1 and CRPA 16-2 decreased to 46% and 34% respectively, when exposed to the concentration of 256 μg/ml ML364. (Figure 1G). To impair the influence of ML364 on *S. aureus* strains, OD_450nm_/OD_600nm_ values were used to quantitate the staphyloxanthin production. At a concentration of 64 μg/ml ML364, the production of staphyloxanthin decreased to 59% in *S. aureus* ATCC 29213 and 52% in MRSA 08-50 (Figure 1H).

### The effects of ML364 treatment on the transcriptome of pathogens

**Figure 2:**
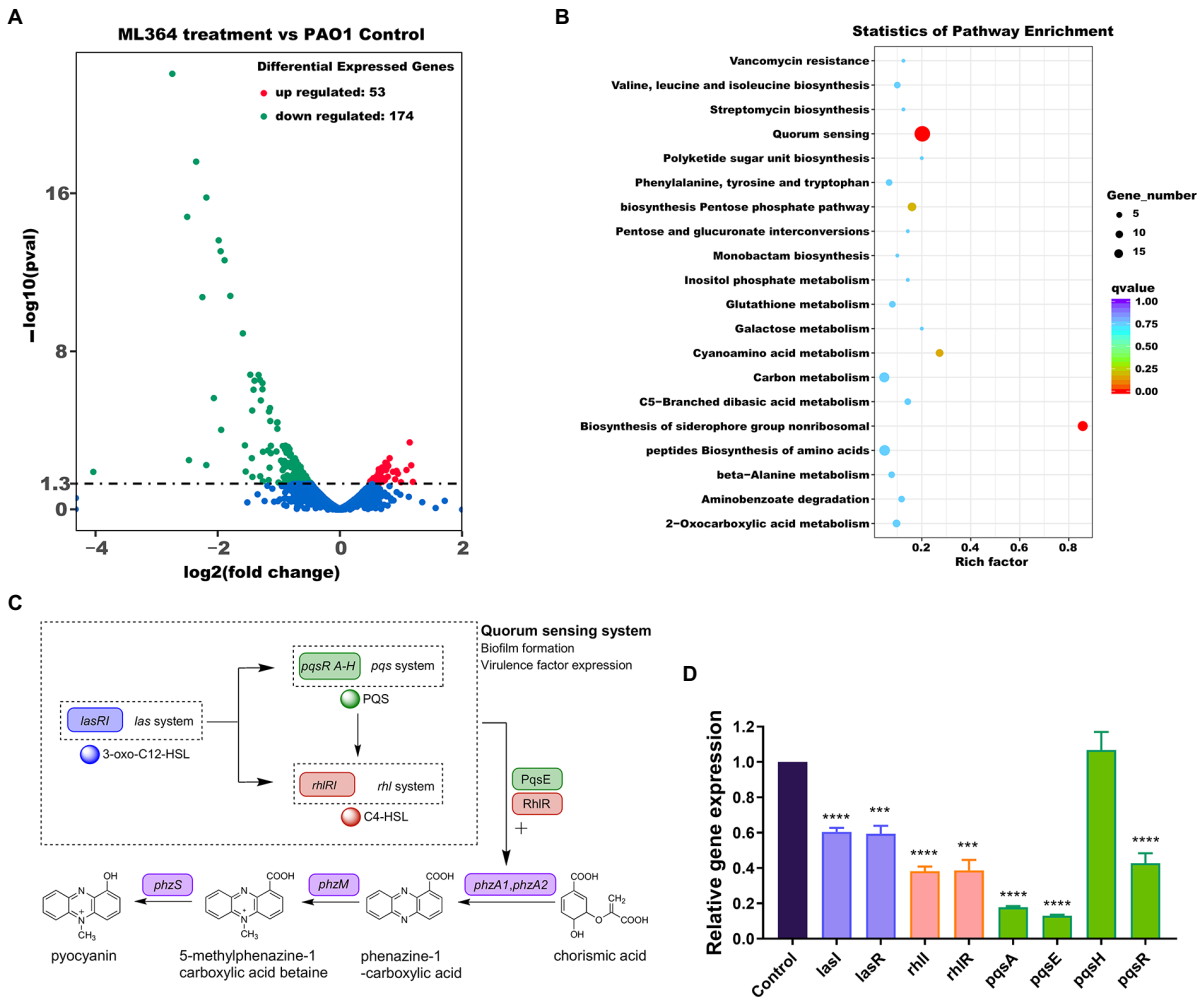
ML364 treatment attenuated virulence factor production by interfering with quorum sensing systems. *Pseudomonas aeruginosa* PAO1 was cultured for 6 h with or without 256 μg/ml ML364. **(A)** Volcano plots of differentially expressed genes in *P. aeruginosa* PAO1 treated with ML364. **(B)** KEGG enrichment analysis of differentially expressed genes in *P. aeruginosa* PAO1 treated with ML364. **(C)** Schematic diagram of the *P. aeruginosa* quorum-sensing hierarchy and biosynthesis system of pyocyanin. **(D)** Transcription levels of quorum sensing-related genes in *P. aeruginosa* PAO1 with ML364 treatment determined by real-time PCR. Data were analyzed *via* the Student’s *t*-test, compared to those of the PAO1 control group; ^*^p < 0.05, ^**^p < 0.01, ^***^p < 0.001, ^****^p < 0.0001.

The mechanisms of action (MoA) behind these effects were explored by transcriptomic sequencing. ML364 treatment of *P. aeruginosa* PAO1 resulted in up-regulation of 53 differentially expressed genes and down-regulation of 174 genes (Figure 2A). And based on the result of KEGG enrichment analysis, the quorum sensing system was mainly disrupted when *P. aeruginosa* PAO1 was treated with ML364 (Figure 2B). Moreover, ML364 treatment has been found to reduce transcription levels of genes associated with QS systems and the virulence factors regulated by QS systems, including rhamnolipid and elastase related genes (Table 1).

**Table 1:** Transcriptomic analysis of quorum sensing-related genes in *Pseudomonas aeruginosa* PAO1 with ML364 treatment.

Gene name	log_2_-fold change	Pval	Description
*hfq*	−0.68317	0.014314	Hfq
*pqsA*	−2.5001	1.56E-15	PqsA
*pqsB*	−2.353	2.50E-18	PqsB
*pqsC*	−2.1859	1.64E-16	PqsC
*pqsD*	−1.9847	2.41E-14	3-oxoacyl-[acyl-carrier-protein] synthase III
*pqsE*	−1.9524	8.59E-14	Quinolone signal response protein
*phnA*	−1.8879	2.45E-13	Anthranilate synthase component I
*phnB*	−1.161	1.11E-05	Anthranilate synthase component II
*rhlI*	−1.0216	3.93E-05	Autoinducer synthesis protein RhlI
*phzA1*	−2.4721	0.0032231	Probable phenazine biosynthesis protein
*phzB1*	−2.2516	1.81E-11	Probable phenazine biosynthesis protein
*phzA2*	−1.9438	9.46E-05	Probable phenazine biosynthesis protein
*phzB2*	−2.187	0.0057322	Probable phenazine biosynthesis protein
*phzM*	−0.89089	0.00059498	Probable phenazine-specific methyltransferase
*phzS*	−1.3098	2.78E-07	Flavin-containing monooxygenase
*lecA*	−1.1774	0.0010461	LecA
*lecB*	−2.7437	8.91E-23	Fucose-binding lectin PA-IIL
*rhlB*	−0.83088	0.0012534	Rhamnosyltransferase chain B
*rhlA*	−1.3313	1.57E-07	Rhamnosyltransferase chain A
*lasB*	−0.84934	0.0006874	Elastase LasB

Log_2_-fold change, log_2_ value of the gene expression level, calculated by comparing the expression levels of ML364-treated and untreated cells. log_2_-fold change > 0, upregulated; log_2_-fold change < 0, downregulated. Pval, *p*-value, *p* < 0.05 means statistical significance.

In bacteria, QS is a the cell-to-cell communication network that can modulate the virulence genes expression and biofilm formation (Waters and Bassler, 2005). The *las* system governs the expression of both the *rhl* and *pqs* systems in the *P. aeruginosa* hierarchy quorum sensing network (Figure 2C; Dong et al., 2021), and the *rhl* system is also under the control of *pqs* systems (Lee and Zhang, 2015). At the same time, PqsE and RhlR could act as regulators of phenazine and pyocyanin production *via* quorum sensing cascade (Higgins et al., 2018). The real-time PCR assay was performed to further verify the effect of ML364 treatment on the expression levels of QS system related genes. In the ML364 treatment group, the expression levels of *lasI*, *lasR*, *rhlI*, *rhlR*, *pqsA*, *pqsE*, and *pqsR* were reduced to 13%–60% when compared to the control group; no noticeable changes were found in the expression levels of *pqsH* (Figure 2D). Furthermore, transcriptome analysis revealed that ML364 treatment also significantly impacted on the transcriptions of quorum sensing-related genes in *S. aureus* ATCC 29213 (Supplementary Table 4).

### The effects of ML364 treatment on DPD/AI-2 response and biofilm formation

**Figure 3:**
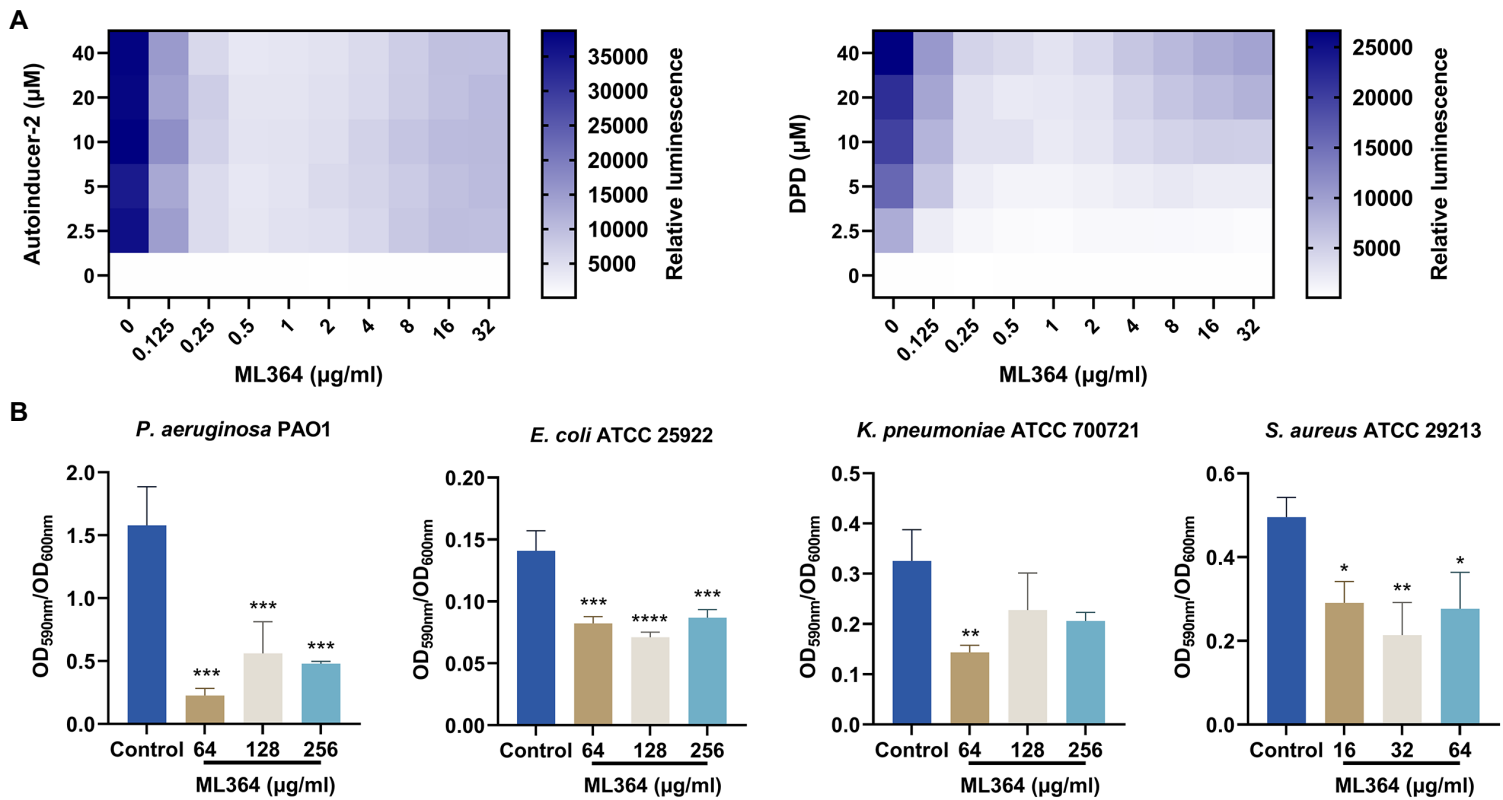
ML364 blocks DPD/AI-2 signaling response and inhibit biofilm formation of different species of bacteria. **(A)** The AI-2 and DPD signaling response of *Vibrio Campbellii* MM32 (LuxN^−^, LuxS^−^) treated with various concentrations of ML364. **(B)** The biofilm quantification of *Pseudomonas aeruginosa* PAO1, *Escherichia coli* ATCC 25922, *Klebsiella pneumoniae* ATCC 700721 and *Staphylococcus aureus* ATCC 29213 treated with ML364. Data were calculated with one-way ANOVA and Bonferroni’s multiple comparisons, compared to those of the control groups; ^*^p < 0.05, ^**^p < 0.01, ^***^p < 0.001, ^****^p < 0.0001.

The DPD/AI-2 signaling of the QS system is the only non-species-specific signaling molecule that could be produced and sensed by both Gram-negative and Gram-positive species (Pereira et al., 2013). We further investigated whether DPD/AI-2 response could be influenced by ML364. In the presence of AI-2 or DPD, bioluminescence produced by *V. Campbellii* MM32 could be significantly inhibited by ML364 treatment. For AI-2 stimulation, 0.5 μg/ml ML364 could exert the maximum inhibitory effect among concentrations from 0.125 μg/ml to 32 μg/ml. And for DPD stimulation, 1 μg/ml ML364 would have the most potent inhibitory effect (Figure 3A).

The biofilm formation ability of bacteria could be influenced by AI-2 signaling transduction. To investigate whether ML364 could exhibit the antivirulence effect across multi-species pathogens, we picked up *P. aeruginosa*, *E. coli*, *K. pneumoniae*, and *S. aureus* from the WHO priority pathogens list to generate the biofilm. The results showed that 64, 128, and 256 μg/ml ML364 could significantly inhibit the biofilm formation of the Gram-negative pathogens, including *P. aeruginosa* PAO1, *E. coli* ATCC 25922, and *K. pneumoniae* ATCC 700721 (Figure 3B). Moreover, 16, 32, and 64 μg/ml ML364 could also significantly inhibit the biofilm formation of *S. aureus* ATCC 29213, the Gram-positive pathogen.

### The analysis of interactions between ML364 and DPD/AI-2 receptors

To understand how ML364 disrupts the AI-2 quorum sensing system, molecular docking and MM/GBSA free energy calculation were applied to investigate the interactions between ML364 and DPD/AI-2 receptors. Since the boron element of AI-2 could not be recognized by Autodock Vina, DPD was used for molecular modeling instead. MM/GBSA free energy calculations showed that ML364, compared with DPD molecule, might have a higher binding affinity with LuxP of *V. Campbellii*, PctA of *P. aeruginosa*, or TlpQ of *P. aeruginosa* (Table 2). The average binding free energy of six types of generalized Born calculations (GB1, GB2, GB5, GB6, GB7, and GB8) between ML364 and LuxP would be −38.11 kcal/mol, while that between DPD and LuxP would be −13.87 kcal/mol. And for PctA and TlpQ protein, the average binding free energy of ML364 would be −26.95 kcal/mol and −24.24 kcal/mol, while that of DPD would be −14.46 kcal/mol and −18.90 kcal/mol.

**Table 2:** MM/GBSA scores (kcal/mol) for the binding poses of the ligands.

Protein	PDB	Ligand	Species	MM/GBSA (kcal/mol)						
				GB1	GB2	GB5	GB6	GB7	GB8	Mean (SD)
LuxP	1JX6	DPD	*Vibrio campbellii*	−19.71	−16.33	−15.99	−4.35	−12.65	−14.18	−13.87 (4.78)
		ML364		−52.45	−41.61	−39.56	−18.28	−40.5	−36.23	−38.11 (10.19)^****^
PctA	5T7M	DPD	*Pseudomonas aeruginosa*	−19.59	−16.85	−17.01	−4.22	−14.13	−14.98	−14.46 (4.90)
		ML364		−30.96	−27.59	−27.62	−19.27	−29.3	−26.98	−26.95 (3.68)^****^
TlpQ	6FU4	DPD	*P. aeruginosa*	−21.61	−18.67	−19.77	−13.72	−22.57	−17.04	−18.90 (2.94)
		ML364		−28.84	−24.85	−24.6	−13.18	−27.05	−26.93	−24.24 (5.15)^**^

The numbers followed by GB are the types of generalized Born calculation (i.e., GB1, GB2, GB5, GB6, GB7, GB8). MM/GBSA scores of ML364-protein complexes were analyzed via the paired *t*-test, compared to that of the corresponding DPD-protein complexes; ^**^p < 0.01, ^****^p < 0.0001.

**Figure 4:**
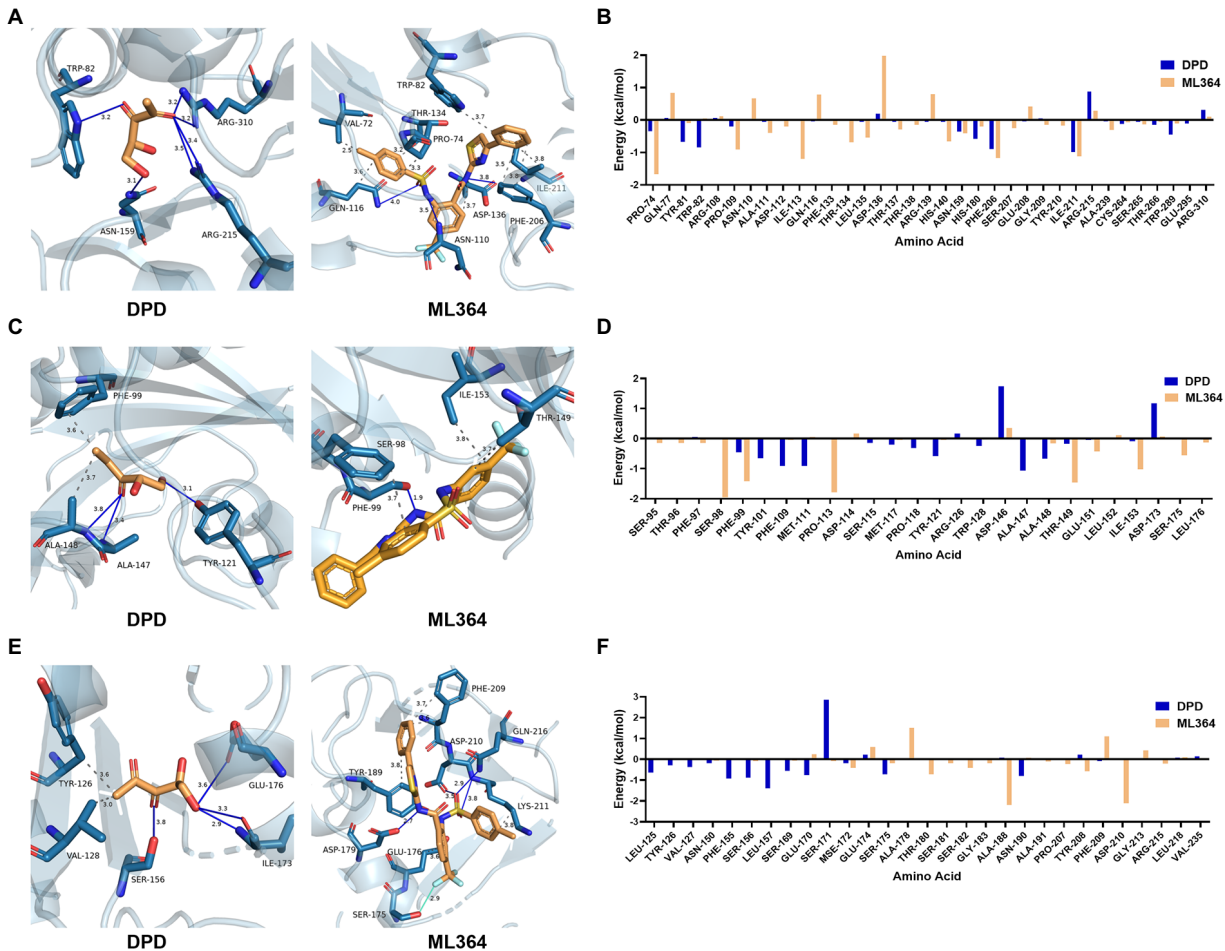
The interactions and per-residue free energy decomposition between DPD or ML364 with DPD/AI-2 receptors. **(A)** The interactions between DPD or ML364 with LuxP protein of *V. Campbellii*. **(B)** The per-residue energy decomposition between LuxP with DPD or ML364. **(C)** The interactions between DPD or ML364 with PctA protein of *Pseudomonas aeruginosa*. **(D)** The per-residue energy decomposition between PctA with DPD or ML364. **(E)** The interactions between DPD or ML364 with TlpQ protein of *P. aeruginosa*. **(F)** The per-residue energy decomposition between TlpQ with DPD or ML364. Protein-ligand interactions are colored as following: blue solid line, hydrogen bond; dash line, hydrophobic interaction; cyan solid line, halogen bond.

The interaction maps showed that DPD might form the conventional hydrogen bond with the tryptophan (position 82), asparagine (position 159), and arginine (position 215 and 310) of LuxP. In contrast, ML364 would form multiple types of interactions with 12 amino acids of LuxP (Figure 4A). The per-residue free energy decomposition analysis showed that ML364 might compete with DPD on several amino acids of LuxP, including the proline at positions 74 and 109, the asparagine at position 159, the phenylalanine at position 206, and the isoleucine at position 211 (Figure 4B). The interaction maps of PctA-ML364 complex and PctA-DPD complex displayed a similar tendency, that ML364 might interact with more amino acids and form more types of interactions when compared with DPD molecule (Figure 4C). Further per-residue energy decomposition analysis showed that ML364 might compete with DPD on phenylalanine (position 99), threonine (position 149) and isoleucine (position 153) of PctA protein (Figure 4D). For TlpQ, the interaction maps showed that ML364 might compete with DPD on glutamic acid (position 176; Figure 4E). Nevertheless, per-residue energy decomposition analysis showed that ML364 might form interactions with markedly different amino acids of the TlpQ when compared with DPD (Figure 4F). And the landscape three-dimension (3D) views of the complexes showed that they occupied two different pockets which are closely in space (Supplementary Figure 1).

### The *in vivo* studies of ML364

**Figure 5:**
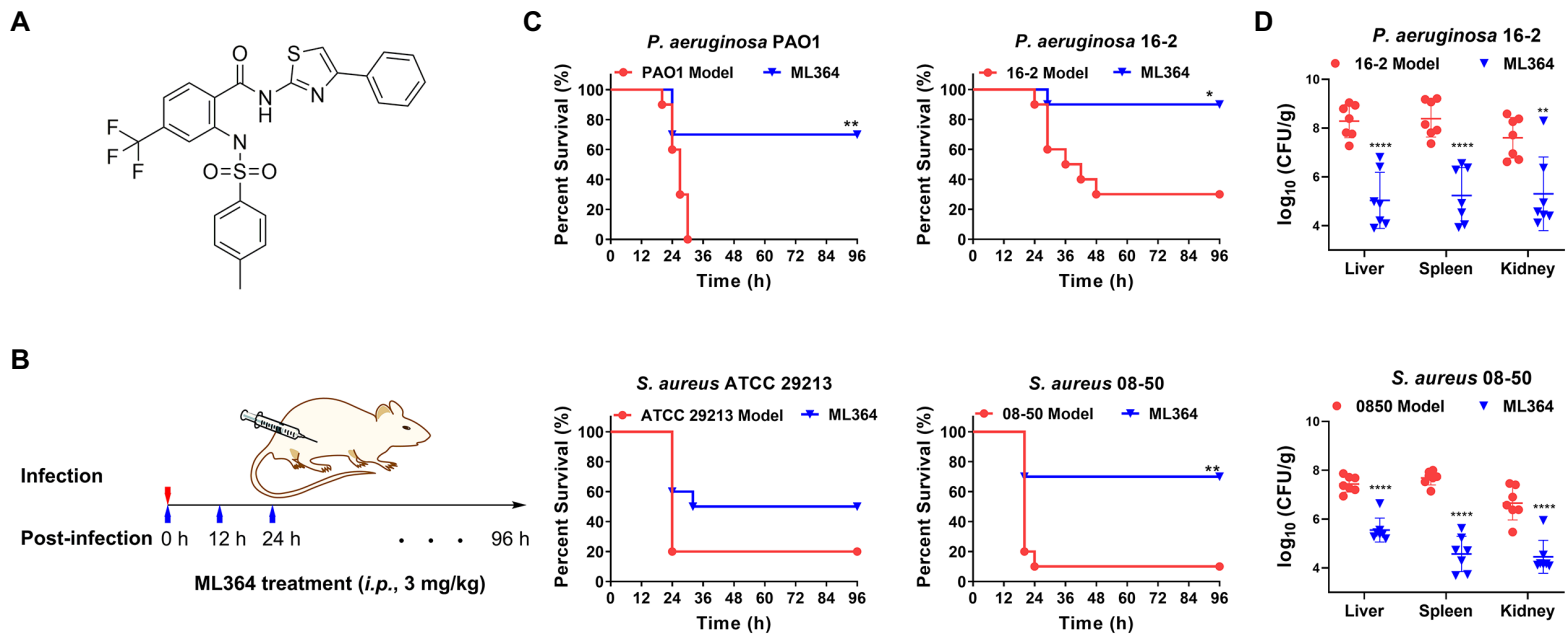
The *in vivo* studies of ML364. **(A)** The chemical structure of ML364. **(B)** The drug administration procedure in systemically infected mice. **(C)** Mice were intraperitoneally infected with *Pseudomonas aeruginosa* PAO1, CRPA 16-2, *Staphylococcus aureus* ATCC 29213, and MRSA 08-50. A dose of 3 mg/kg ML364 was intraperitoneally injected at 0, 12, and 24 h post-infection. Survival rates of mice were monitored until 4 days post-infection. **(D)** Bacterial loads in organs (liver, spleen, and kidney) influenced by ML364 therapy in the CRPA 16-2 and MRSA 08-50 infection model counted at 26 h post-infection (*n* = 7 per group). ^*^p < 0.05, ^**^p < 0.01, ^****^p < 0.0001 (Compared with the model group).

The maximum tolerated dose (MTD) of ML364 in mice was first determined (Supplementary Figure 2). The results showed that the MTD of ML364 was greater than or equal to 100 mg/kg, when ICR mice received an intraperitoneal injection (*i.p.*). Furthermore, the compound under 100 mg/kg could not affect the body weight gain of mice. Consequently, 3 mg/kg (*i.p.*) could be considered as a safe dose for the *in vivo* study (Figures 5A,B). The survival rates of systemically infected mice were significantly higher when treated with ML364, compared to the control group. In the *P. aeruginosa* PAO1 or the CRPA 16-2 infection mouse model, ML364 treatment could significantly improve the survival rate from 0% to 70% or from 30% to 90%, respectively. The survival rates of mice infected with *S. aureus* ATCC 29213 or MRSA 08-50 could be increased from 20% to 50% or even from 10% to 70%, by the administration of ML364 (Figure 5C).

Furthermore, we investigated whether ML364 treatment could also decrease bacterial loads in systemically infected mice due to its antivirulence effect *in vivo*. Colony counting was used to determine the bacterial loads in the liver, spleen, and kidney of mice. In Figure 5D, ML364 was able to significantly reduce the bacterial loads in the organs of mice (*p* ≤ 0.01), when the mice were systemically infected with CRPA 16-2 or MRSA 08-50. In the ML364 treatment groups, average CFU counts were reduced by more than one order of magnitude.

## Discussion

For now, the broad-spectrum antibacterials may hardly meet the clinical needs due to the rapid emergence of antimicrobial resistance. So new strategies have been developed to against the lacking of antibacterials with new mechanism of action (MoA). Rather than directly killing bacteria like traditional bactericides, antivirulence strategies indirectly kill the pathogens, which leads to the reducing evolutionary pressure for bacteria. By attenuating the virulence, the pathogenicity of bacteria would be decreased, and then the host immune system could eliminate the established infections (Johnson and Abramovitch, 2017). For now, most antivirulence agents in the pipeline have a narrow antibacterial spectrum and limited indications. Our main purpose is to develop broad-spectrum antivirulence agents that could target on both Gram-positive and Gram-negative pathogens. Furthermore, we hope these broad-spectrum antivirulence agents could possess a novel scaffold and could be optimized to the new lead compounds. Fortunately, we found ML364 could inhibit the productions of both pyocyanin of *Pseudomonas aeruginosa* and staphyloxanthin of *Staphylococcus aureus in vitro*. Although the growth curves of some *S. aureus* strains would be depressed by ML364 treatment, the MICs of ML364 for these strains were still above 512 μg/ml and the morphology of the strains could not be changed by ML364 treatment.

Further transcriptome sequencing and KEGG enrichment analysis showed that the quorum sensing systems of both *P. aeruginosa* and *S. aureus* were mainly disrupted by ML364 treatment. It reminded us that ML364 might interfere with the common signaling of QS systems which could be recognized by both *P. aeruginosa* and *S. aureus*. Quorum sensing is a cell–cell communication mechanism in which bacteria produce, release and detect signaling molecules (Waters and Bassler, 2005). In *P. aeruginosa*, the transcriptions of genes in many other quorum sensing systems, including *las*, *pqs*, and *rhl* systems, could also be significantly inhibited by ML364 treatment. Although the transcription of *PqsH* could not be influenced by ML364 treatment, the transcriptions of many other genes in *pqs* systems could be inhibited by ML364, including *pqsR*, *pqsA*, *pqsB*, *pqsC*, *pqsD*, and *pqsE*. The relationship between these quorum sensing systems and AI-2 signaling is still needed for further investigation. The non-species-specific quorum sensing signaling could be seen as the worldwide broadcast among inter-species, including autoinducer-3 (AI-3, Gram-negative), non-species specific DSF signaling (Gram-negative) and autoinducer-2 (AI-2, Gram-negative and Gram-positive; Pereira et al., 2013). To date, AI-2 of the QS system is the only non-species-specific signaling molecule responsible for the cross-talk between Gram-negative and Gram-positive species (Wu et al., 2020). So we further investigated whether ML364 could influence the response for DPD/AI-2 signaling. Genetically modified strains have been developed to detect QSI molecules by expressing reporter genes in response to specific QS signals (Saurav et al., 2017). Due to the absence of the LuxN receptor which needed to respond to AI-1, and carried mutations of luxS which could catalyze the biosynthesis of DPD, *V. Campbellii* MM32 (*LuxN*^−^, *LuxS*^−^ was used as a reporter for AI-2 signaling sensing). Our study showed that ML364 could significantly inhibit the sensing of DPD/AI-2 signaling in *V. Campbellii* MM32. Moreover, the biofilm could be generated by multiple species of bacteria and regulated by AI-2 signaling (Pereira et al., 2013). And we further uncovered that biofilm formation could also be inhibited by ML364 treatment across multi-species pathogens, including *P. aeruginosa*, *E. coli*, *K. pneumoniae*, and *S. aureus*. This finding reminded us that ML364 might interfere with AI-2 quorum sensing system to against broad-spectrum pathogens. And the results of cytotoxicity showed that less than or equal 512 μg/ml of ML364 had no influence on the variability of the Vero cells (Supplementary Figure 3).

Subsequently, molecular modeling was applied to investigate whether ML364 could interact with the receptors of DPD/AI-2. Because AI-2 receptor of *S. aureus* is still unknown, LuxP of *V. Campbellii*, PctA of *P. aeruginosa* or TlpQ of *P. aeruginosa* were chosen for *in silico* study. Since it could provide greater accuracy compared to the majority of scoring functions for docking, end-point binding free energy calculation with MM/GBSA has become the most popular method for binding free energy prediction (Wang et al., 2019). FastDRH webserver could predict the binding modes of ligands by the integrated structure-truncated MM/GBSA rescoring procedures (Wang et al., 2022). The results of molecular docking and MM/GBSA free energy prediction showed that ML364, compared with DPD molecule, might have higher binding affinity with the receptors of DPD/AI-2. By summing the interactions between all residues and ligands in the system, the per-residue energy decomposition calculates the energy contribution of single residues. And further per-residue energy decomposition analyses confirmed that ML364 might compete with DPD to bind these proteins. Due to the receptor-ligand complex structure would be minimized before rescoring and hotspot analysis, there will be slight differences between the results of the interaction maps with that of per-residue energy decomposition analyses.

DPD could be generally synthesized by the enzyme LuxS and spontaneously interconvert into AI-2 signaling molecules. LuxS homologs could be identified in 537 of the 1,402 bacterial genomes currently sequenced, including *Streptococcus* spp. (Lyon et al., 2001), *Staphylococcus* spp. (Xu et al., 2006), *Salmonella* spp. (Surette et al., 1999), *Klebsiella* spp. (Zhu et al., 2011). However, *P. aeruginosa* could not produce AI-2, but it could robustly respond to this signaling molecule by the PctA and TlpQ receptors (Zhang et al., 2020). In our study, ML364 could still inhibit the virulence of *P. aeruginosa* in the absence of DPD/AI-2. And the MM/GBSA energy calculations showed that ML364 might directly bind to the receptors of AI-2. These results reminded us that ML364 might play the role of inverse agonist for the AI-2 receptors in *P. aeruginosa*. An inverse agonist is a ligand that binds to the same receptor-binding site as an agonist and not only antagonizes the effects of an agonist but also produces a biological response opposite to that of the agonist (Kenakin, 2004). And the further investigation will be needed to explore the detailed interactions between ML364 and the AI-2 receptors.

Finally, ML364 significantly improved the survival rate of systemically infected mice and attenuate the bacterial loads in their organs. The results of the *in vivo* study suggested that ML364 might diminish the pathogenicity of bacteria and then the host immune system could eliminate the pathogens *in vivo*. Moreover, we evaluated several analogs of ML364, which named by their CAS Registry Numbers, by autoinducer-2 inhibition assay. And the results showed that analogs 378,228-61-6 and 380,167-69-1, which possessed the same scaffold with ML364, could also inhibit the bioluminescence produced by *V. Campbellii* MM32 (Supplementary Figure 4). It reminded us that these analogs might also be the potential AI-2 inhibitors. In conclusion, the novel scaffold small-molecule ML364 could exert broad-spectrum antivirulence effect both *in vitro* and *in vivo* by interfering with quorum sensing system of the pathogens. This finding might shed light on the discovery of nontraditional antibacterials against antimicrobial resistance.

## Data availability statement

The data presented in the study are deposited in the BioProject database at NCBI repository, accession number PRJNA833549. The deposited data had been released to the public at: https://www.ncbi.nlm.nih.gov/bioproject/PRJNA833549.

## Ethics statement

The animal study was reviewed and approved by Laboratory Animal Welfare and Ethics Committee of the Institute of Medicinal Biotechnology, Peking Union Medical College.

## Author contributions

YZ, LD, and XY designed the study. YZ and LD performed the experiments. YZ, LD, LS, XH, XW, TN, XLu, JP, XLi, PW, PP, and KY analyzed the data. YZ, LD, KY, and XY prepared the manuscript with input and approval from all authors. All authors contributed to the article and approved the submitted version.

## Funding

This work was supported by the National Natural Science Foundation of China (grant numbers 82104248, 32141003, 82204466, and 82104249) and the CAMS Innovation Fund for Medical Sciences (CIFMS) (grant numbers 2021-1-12M-030 and 2021-1-12M-039).

## Conflict of interest

The authors declare that the research was conducted in the absence of any commercial or financial relationships that could be construed as a potential conflict of interest.

## Publisher’s note

All claims expressed in this article are solely those of the authors and do not necessarily represent those of their affiliated organizations, or those of the publisher, the editors and the reviewers. Any product that may be evaluated in this article, or claim that may be made by its manufacturer, is not guaranteed or endorsed by the publisher.

## Supplementary material

The Supplementary material for this article can be found online at: https://www.frontiersin.org/articles/10.3389/fmicb.2022.980217/full#supplementary-material
