# Supplementary material for: ML364 exerts the broad-spectrum antivirulence effect by interfering with the bacterial quorum sensing system
Source: Front Microbiol. 2022 Dec 22;13:980217. doi: 10.3389/fmicb.2022.980217 (PMC9813848; doi:10.3389/fmicb.2022.980217)
Supplement: Supplementary file 1 [file Data_Sheet_1.docx]

Supplementary Material

ML364 exerts the broad-spectrum antivirulence effect by interfering with the bacterial quorum sensing system

Youwen Zhang^1, †, *^, Limin Dong^2, †^, Lang Sun^1^, Xinxin Hu^1^, Xiukun Wang^1^, Tongying Nie^1^, Xue Li^1^, Penghe Wang^1^, Pengbo Pang^1^, Jing Pang^1^, Xi Lu^1^, Kaihu Yao^2^, Xuefu You^1, *^

^1^Beijing Key Laboratory of Antimicrobial Agents, Institute of Medicinal Biotechnology, Chinese Academy of Medical Sciences & Peking Union Medical College, Beijing, China

^2^Key Laboratory of Major Diseases in Children, Ministry of Education, National Key Discipline of Pediatrics (Capital Medical University), Beijing Pediatric Research Institute, Beijing Children’s Hospital, Capital Medical University, National Center for Children’s Health, Beijing, China

^†^ These authors have contributed equally to this work and share first authorship.

*** Correspondence:**Youwen Zhang, [youwenzhang@imb.cams.cn](mailto:youwenzhang@imb.cams.cn)
Xuefu You, xuefuyou@imb.pumc.edu.cn

# Supplementary Figures and Tables

## Supplementary Figures

**

**

**Supplementary Figure 1.** The 3D views of the best docking poses of ML364 or DPD within the receptors of DPD/AI-2. A. The docking poses of DPD and ML364 for LuxP of *V. campbellii*. B. The docking poses of DPD and ML364 for PctA of *P. aeruginosa*. C. The docking poses of DPD and ML364 for TlpQ of *P. aeruginosa*.





**Supplementary Figure 2.** The influence of ML364 on the body weight of ICR mice. Mice were intraperitoneally injected with 100mg/kg ML364 (n=10 per group).


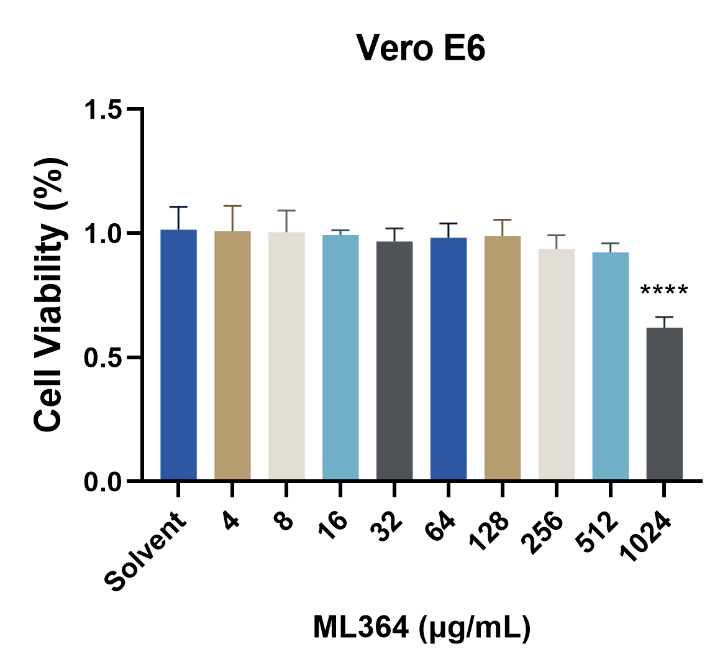


**Supplementary Figure 3.** The cytotoxicity of ML364 on Vero-E6 cells. Data were calculated with one-way ANOVA and Bonferroni’s multiple comparisons, in comparison to the solvent group; *****P* < 0.0001.


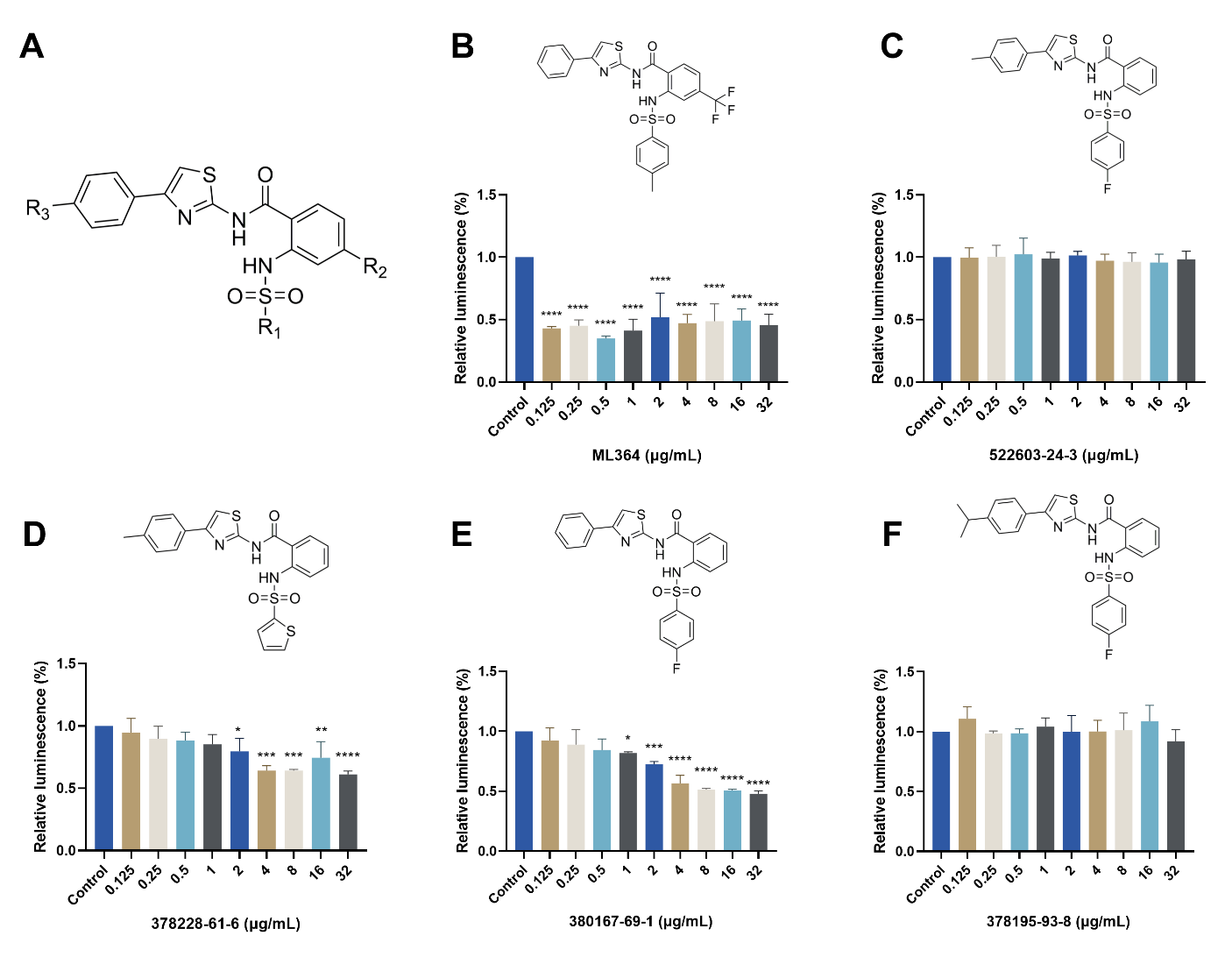


**Supplementary Figure 4.** The DPD (10 μM) signaling response of *V. Campbellii* MM32 (*LuxN*^-^, *LuxS*^-^) treated with various concentrations of ML364 and its analogues. A. Markush structure of ML364 and its analogues. B. The relative bioluminescence (%) changes influenced by ML364 treatment. C. The relative bioluminescence (%) changes influenced by ML364 analogue (CAS: 522603-24-3). D. The relative bioluminescence (%) changes influenced by ML364 analogue (CAS: 378228-61-6). E. The relative bioluminescence (%) changes influenced by ML364 analogue (CAS: 380167-69-1). F. The relative bioluminescence (%) changes influenced by ML364 analogue (CAS: 378195-93-8). Data were calculated with one-way ANOVA and Bonferroni’s multiple comparisons, in comparison to those of the control group; **P* < 0.05, ***P* < 0.01, ****P* < 0.001, *****P* < 0.0001.

## Supplementary Tables

**Supplementary Table 1.** Primers for Real-Time PCR used in this study

| **Gene** | **Type** | **Oligonucleotide sequence (5’-3’)** | **Reference** |
| --- | --- | --- | --- |
| *oprL* | Fw | CCAACAGCGGTGCCGTTGA | This study |
|  | Rev | GCCATATTGTACTCGCGGGT |  |
| *lasI* | Fw | ATGATCGTACAAATTGGTCGGCGCG | (Dong et al., 2019) |
|  | Rev | CGCTCCTTGAACACTTGAG |  |
| *lasR* | Fw | CTGTGGATGCTCAAGGACTAC | (El-Mowafy et al., 2014) |
|  | Rev | AACTGGTCTTGCCGATGG |  |
| *rhlI* | Fw | CGGCATCAGGTCTTCATCG | (El-Mowafy et al., 2014) |
|  | Rev | GTAGCGGGTTTGCGGATG |  |
| *rhlR* | Fw | CGGTCTGCCTGAGCCATC | (El-Mowafy et al., 2014) |
|  | Rev | GCCAGCGTCTTGTTCGG |  |
| *pqsH* | Fw | GCGCGGATCGAGTTCATC | (Viducic et al., 2016) |
|  | Rev | CAGGGCGATTCCCACTGA |  |
| *pqsE* | Fw | GGATGCCGAATTGGTTTG | (Dong et al., 2019) |
|  | Rev | GGTCGTAGTGCTTGTGGG |  |
| *pqsR* | Fw | ATCGACGAGGAACTGAAGA | (El-Mowafy et al., 2014) |
|  | Rev | CTGATCTGCCGGTAATTGG |  |
| *pqsA* | Fw | GACCGGCTGTATTCGATTC | (El-Mowafy et al., 2014) |
|  | Rev | GCTGAACCAGGGAAAGAAC |  |

**Supplementary Table 2.** Antibiogram for ML364

| **Specie** | **Isolate** | **Phenotype** | **MIC (μg/mL)** | |
| --- | --- | --- | --- | --- |
|  |  |  | **Levofloxacin** | **ML364** |
| *Staphylococcus epidermidis* | ATCC 12228 | MSSE | 0.25 | >512 |
|  | 16-4 | MSSE | 0.12 | 512 |
|  | 16-5 | MRSE | 8 | 512 |
| *Staphylococcus aureus* | ATCC 29213 | MSSA | 0.12 | >512 |
|  | ATCC 33591 | MRSA | 0.12 | >512 |
|  | 15 | MSSA | 0.06 | >512 |
|  | 08-50 | MRSA | 0.12 | >512 |
|  | 16-1 | MSSA | 0.12 | >512 |
|  | 16-30 | MRSA | 32 | >512 |
| *Enterococcus faecalis* | ATCC 29212 | VSE | 1 | >512 |
|  | ATCC 51299 | VRE | 0.5 | >512 |
|  | ATCC 51575 | VRE | 0.5 | >512 |
|  | 16-6 | VSE | 1 | >512 |
| *Enterococcus faecium* | ATCC 700221 | VRE | 32 | >512 |
|  | 16-5 | VSE | 64 | >512 |
|  | 12-1 | VRE | 32 | >512 |
| *Escherichia coli* | ATCC 25922 | ESBLs(-) | ≤0.03 | >512 |
|  | ATCC 35218 | ESBLs(+) | 0.06 | >512 |
|  | ATCC 2469 | NDM-1(+) | 8 | >512 |
|  | 16-1 | ESBLs(+) | 0.5 | >512 |
|  | 16-7 | ESBLs(-) | 0.5 | >512 |
| *Klebsiella pneumoniae* | ATCC 700603 | ESBLs(+) | 1 | >512 |
|  | ATCC BAA-2146 | NDM-1(+) | >128 | >512 |
|  | 16-14 | ESBLs(+) | 0.5 | >512 |
| *Pseudomonas aeruginosa* | ATCC 27853 |  | 2 | >512 |
|  | PAO1 |  | 4 | >512 |
|  | 16-2 | CRPA | 16 | >512 |
|  | 16-11 |  | 0.5 | >512 |
| *Acinetobacter baumannii* | ATCC 19606 |  | 0.25 | >512 |
| *Enterobacter cloacae* | ATCC 43560 |  | ≤0.03 | >512 |
| *Enterobacter aerogenes* | ATCC 13048 |  | 0.06 | >512 |
| *Serratia marcescens* | ATCC 21074 |  | 0.12 | >512 |
| *Citrobacter freundii* | ATCC 43864 |  | 0.12 | >512 |
| *Providentia rettgeri* | ATCC 31052 |  | 0.12 | >512 |
| *Proteus vulgaris* | ATCC 29905 |  | ≤0.03 | >512 |
| *Proteus mirabilis* | ATCC 49565 |  | 0.06 | >512 |
| *Stenotrophomonas maltophilia* | ATCC 13636 |  | 4 | >512 |
| *Shigella flexneri* | ATCC 12022 |  | ≤0.03 | >512 |

MIC, minimum inhibitory concentration; MSSE, methicillin-sensitive *Staphylococcus epidermidis*; MRSE, methicillin-resistant *Staphylococcus epidermidis*; MSSA*,* methicillin-sensitive *Staphylococcus aureus*; MRSA, methicillin-resistant *Staphylococcus aureus*; VISA, vancomycin-intermediate *Staphylococcus aureus*; VSE, vancomycin-susceptible *Enterococcus*; VRE, vancomycin-resistant *Enterococcus*; ESBL, extended-spectrum beta-lactamase; NDM-1, New Delhi metallo-beta-lactamase 1; CRPA, carbapenem-resistant *Pseudomonas aeruginosa*; CRAB, carbapenem-resistant *Acinetobacter baumannii*.

**Supplementary Table 3.** Checkerboard assay results of MEM/MET-based combination with ML364

| **Species** | **Drugs** | **MIC (μg/mL)** | |
| --- | --- | --- | --- |
|  |  | **Alone** | **Combination** |
| CRPA 16-2 | MEM | 16 | 16 |
|  | ML364 | ＞512 | ＞512 |
| MRSA 0850 | MET | 1024 | 1024 |
|  | ML364 | ＞64 | ＞64 |

MEM, meropenem; MET, methicillin.

**Supplementary Table 4.** Effect of ML364 treatment on the transcription of quorum sensing-related genes in *S. aureus* ATCC 29213

| **Gene name** | **log_2_Fold Change** | **Pval** | **Description** |
| --- | --- | --- | --- |
| *lacD* | -4.840942631 | 2.10E-96 | tagatose-bisphosphate aldolase |
| *sspA* | 2.591547275 | 4.65E-43 | Glu-specific serine endopeptidase SspA |
| *secDF* | 1.163544772 | 2.85E-20 | protein translocase subunit SecDF |
| *oppA* | 1.31817264 | 6.36E-18 | peptide ABC transporter substrate-binding protein |
| *oppF_2* | 1.324691445 | 1.25E-11 | ATP-binding cassette domain-containing protein |
| *secA2* | -1.002675523 | 2.55E-11 | accessory Sec system translocase SecA2 |
| *gsiB* | 0.763498184 | 1.99E-09 | ABC transporter substrate-binding protein |
| *oppD_2* | 1.152993148 | 5.00E-07 | ABC transporter ATP-binding protein |
| *ffh* | 0.495758615 | 1.60E-05 | signal recognition particle protein |
| *secA* | 0.523344248 | 4.72E-05 | preprotein translocase subunit SecA |
| *ftsY* | 0.655180982 | 8.02E-05 | signal recognition particle-docking protein FtsY |
| *nikB* | 1.009089528 | 0.000429388 | ABC transporter permease |
| *toxF* | -0.990230393 | 0.000915132 | DMT family transporter |
| *gsiC* | -1.377482736 | 0.001247992 | ABC transporter permease |
| *rpfB* | -0.490731823 | 0.003674744 | acyl--CoA ligase |
| *gsiA* | 1.027439959 | 0.004032413 | ABC transporter ATP-binding protein |
| *hfq* | -0.552067636 | 0.006591293 | RNA chaperone Hfq |
| *comK* | -1.751710308 | 0.017126424 | competence protein ComK |
| *DQV20_10110* | 0.932639065 | 0.02285521 | ABC transporter permease |
| *agrA* | -1.280764112 | 0.025710624 | response regulator transcription factor |
| *oppC* | 0.586481738 | 0.031112536 | ABC transporter permease |
| *secG* | -0.338783564 | 0.043839574 | preprotein translocase subunit SecG |

Log_2_-fold Change: log_2_ value of the gene expression level, calculated by comparing the values of the ML364-treated group to those of the untreated control group. log_2_-fold change > 0, upregulated; log_2_-fold change < 0, downregulated. Pval, P-value, *P*<0.05 means statistical significance.

**References (additional to those included in the main text)**

Dong, L., Pang, J., Wang, X., Zhang, Y., Li, G., Hu, X., et al. (2019). Mechanism of pyocyanin abolishment caused by mvaT mvaU double knockout in Pseudomonas aeruginosa PAO1. *Virulence* 11(1)**,** 57-67. doi: 10.1080/21505594.2019.1708052.

El-Mowafy, S.A., Abd El Galil, K.H., El-Messery, S.M., and Shaaban, M.I. (2014). Aspirin is an efficient inhibitor of quorum sensing, virulence and toxins in Pseudomonas aeruginosa. *Microb Pathog* 74**,** 25-32. doi: 10.1016/j.micpath.2014.07.008.

Viducic, D., Murakami, K., Amoh, T., Ono, T., and Miyake, Y. (2016). RpoN Modulates Carbapenem Tolerance in Pseudomonas aeruginosa through Pseudomonas Quinolone Signal and PqsE. *Antimicrob Agents Chemother* 60(10)**,** 5752-5764. doi: 10.1128/aac.00260-16.
